# Adalimumab (ADA) in Pediatric Non-infectious Uveitis: An Observational Study

**DOI:** 10.7759/cureus.59019

**Published:** 2024-04-25

**Authors:** Rihab A Ghanma, Laura Steeples, Sasa Pockar, Vinod Sharma, Alice Chieng, Jane Ashworth

**Affiliations:** 1 Uveitis/Ophthalmology, University of Manchester, Manchester, GBR; 2 Medical Retina and Uveitis/Ophthalmology, Royal Medical Services, Amman, JOR; 3 Paediatric Rheumatology, University of Manchester, Manchester, GBR; 4 Paediatric Ophthalmology, University of Manchester, Manchester, GBR

**Keywords:** anti-drug antibodies, paediatric non-infectious uveitis, steroids, methotrexate, adalimumab

## Abstract

Objectives: Pediatric uveitis is a rare but sight-threatening condition. Prompt and adequate treatment is crucial to preserve vision and avoid long-term complications. In cases that are resistant to corticosteroids and disease-modifying anti-rheumatic drugs (DMARDs), anti-tumor necrosis (anti-TNF) biologic agents are usually added. In this study, we report our experience with adalimumab (ADA) anti-TNF use in this group of patients.

Methods: This is a retrospective observational study conducted in a tertiary pediatric uveitis clinic, in Manchester Royal Eye Hospital. All patients were pediatric patients (aged 2-18 years old) under follow-up during the period of six months. The patients’ data were analyzed according to the diagnosis, age of onset of uveitis, systemic medications used before and concomitantly with ADA, duration of uveitis before starting ADA, its effect, and time to notice the therapeutic effect in controlling inflammation. Finally, cases were reviewed for the development of anti-drug antibodies.

Results: Forty-two patients were included in the study. Idiopathic uveitis was diagnosed in 47.6% of patients and 40.5% of patients were associated with juvenile idiopathic arthritis (JIA). Most (97.6%) of patients were using topical steroids before starting ADA and 95.2% continued using steroids after established ADA use, but systemic steroid use was reduced from 33.3% to 14.3%. The most common non-biologic DMARD used before ADA was methotrexate (MTX) (90.5%). One-third of the patients started ADA between 6 and 12 months after the diagnosis of uveitis, while this percentage dropped to 9.5% the year after diagnosis. Seventy-eight percent of patients acquired complete clinical control of inflammation on ADA use. Almost 78.6% of patients showed a full response in less than six months. In eight patients who were not controlled or were transiently controlled on ADA, three patients had positive anti-drug antibodies. In one patient, antidrug antibodies were identified after 12 years of ADA use, and in another, after 4 years.

Conclusion: Adalimumab is an effective, well-tolerated drug in children with uveitis refractory to non-biologic DMARD therapy. DMARDs were usually used alongside ADA in this cohort and few patients had confirmed ADA antibodies.

## Introduction

Pediatric uveitis is a rare but sight-threatening condition. Prompt and adequate treatment is crucial to preserve vision and avoid long-term complications. Corticosteroids and disease-modifying anti-rheumatic drugs (DMARDs) are the first-line therapy to treat non-infectious uveitis in children. In cases resistant to these therapies, anti-tumor necrosis factor (anti-TNF) agents are usually added [1-2]. Adalimumab (ADA) is a recombinant human IgG1 monoclonal antibody against human TNF-α. It was approved in 2016 by the US Food and Drug Administration as the first systemic anti-TNF-α agent for treating non-infectious intermediate, posterior, and pan uveitis [1-2].

## Materials and methods

This is a record-based observational study conducted in a tertiary pediatric uveitis clinic, at the Manchester Royal Eye Hospital in the United Kingdom. We reviewed in detail the electronic files of all the pediatric patients (between 2 and 18 years old) who were under follow-up for six months (March 2022-September 2022) in the pediatric uveitis clinic and included all patients using ADA. The patients’ data were analyzed according to the diagnosis, age of onset of uveitis, systemic medications since the diagnosis, and the medications used before starting and along with ADA, along with the duration of the disease before administering ADA.

The ADA was specifically studied for its therapeutic effect and in patients with a positive response, the time to achieve therapeutic effect in controlling inflammation. Furthermore, we recorded treatment failures: firstly, transiently controlled cases (who got uveitis reactivation after quiescence for more than three months); and secondly, cases refractory to ADA, where disease control was not achieved within 6 months of ADA initiation. Finally, we examined the records of those who developed anti-drug antibodies. 

We excluded patients with treatment non-compliance, including not following the planned protocol of the injections, tablets, or drops or medication interruptions (intentional non-compliance or justification for side effects of medications). The study was a retrospective (record-based) study carried out according to the Declaration of Helsinki. No names were used for data collection, and confidentiality was ensured at all levels.

## Results

**Figure 1:**
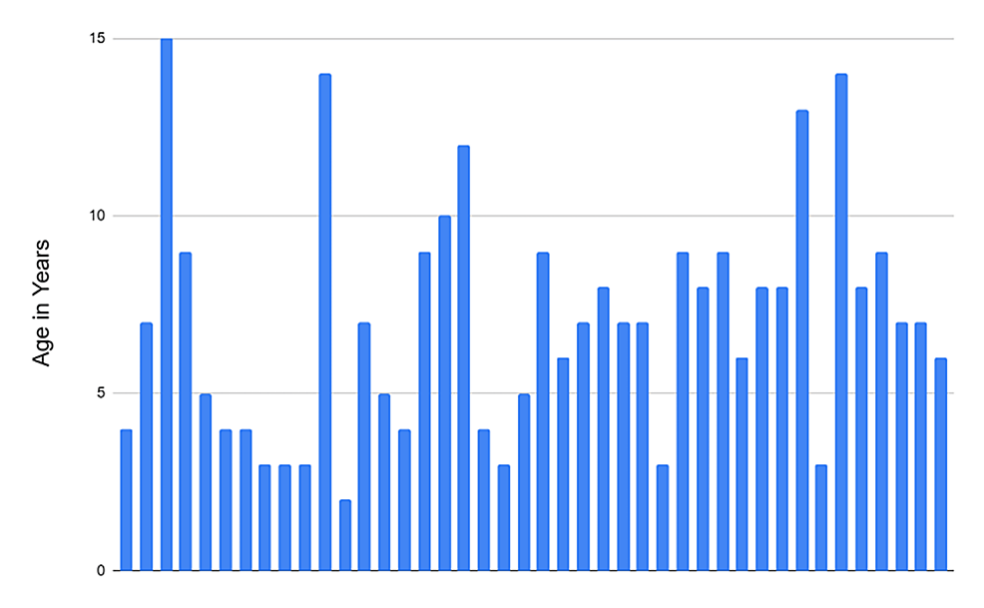
Age of onset of uveitis for adalimumab users in years.

Forty-two patients were included in the study: 30 females and 12 males. More than a third were diagnosed before the age of five years old, while 12% of the patients had uveitis diagnosed after the age of 10 years. The highest age group in number was those between 7(16.7%) and 9(14.3%) years, forming 43% of the total (Figure 1).

**Figure 2:**
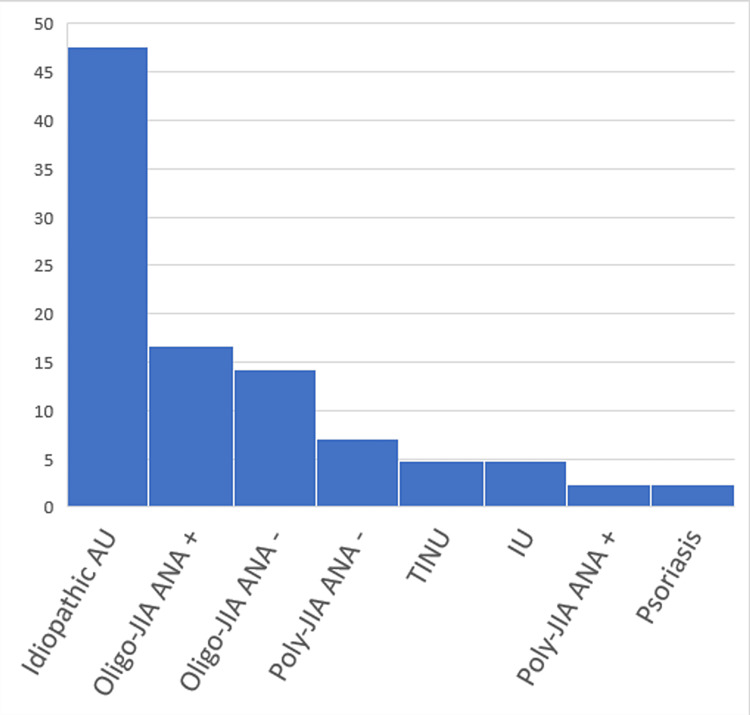
Uses of adalimumab in the pediatric uveitis clinic.

The etiology of uveitis was idiopathic in 47.6% of cases. Just above forty percent (40.5%) were associated with Juvenile Idiopathic Arthritis (JIA). The largest group of the latter was the oligoarticular JIA with positive antinuclear antibody (ANA) (16.7%), followed by oligoarticular JIA with negative ANA. The rest were polyarticular (9.5%). The remaining were 4.8% diagnosed with Tubulointerstitial Nephritis and Uveitis Syndrome (TINU), and the same percentage with idiopathic Intermediate uveitis (IU), and 2.4% had psoriasis (Figure 2).

**Figure 3:**
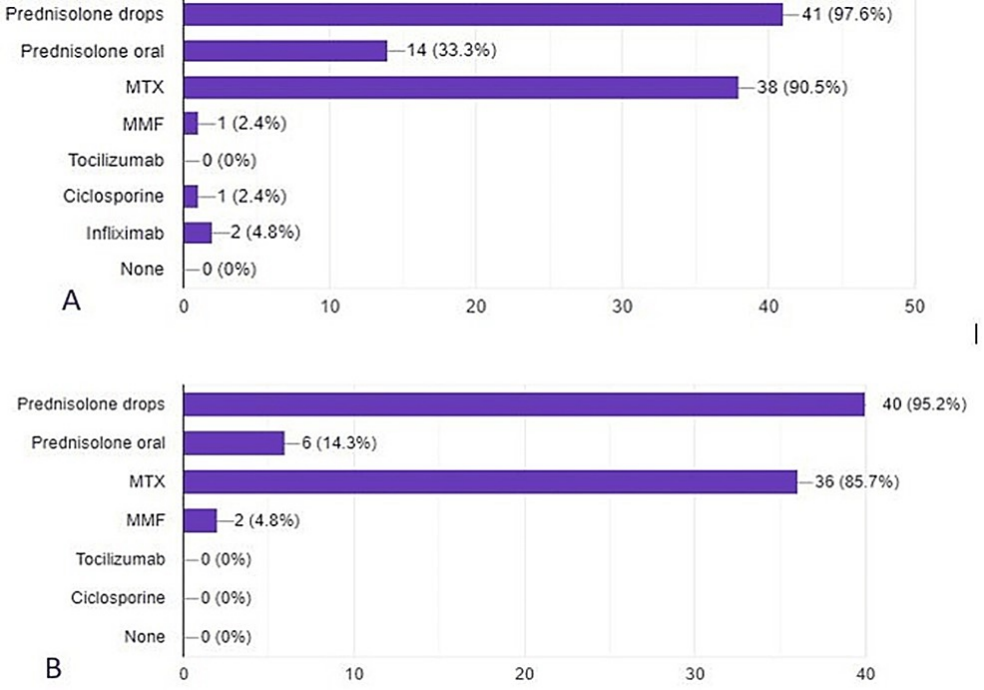
A: Medications used for uveitis before introducing ADA. B: Medications used along with ADA for uveitis. ADA, adalimumab

Almost 97.6% of the patients were using topical steroids before starting ADA, and 95.2% continued using them after establishing ADA use. However, systemic steroid use was reduced from 33.3% to 14.3% when ADA was added. The most common immunomodulatory drug (DMARDs) used before ADA was methotrexate (MTX) (90.5%). MTX use was only slightly reduced to 85.7% after starting ADA. Mycophenolate mofetil was used only by two patients with ADA (Figure 3). Only three patients were on ADA monotherapy systemically.

**Figure 4:**
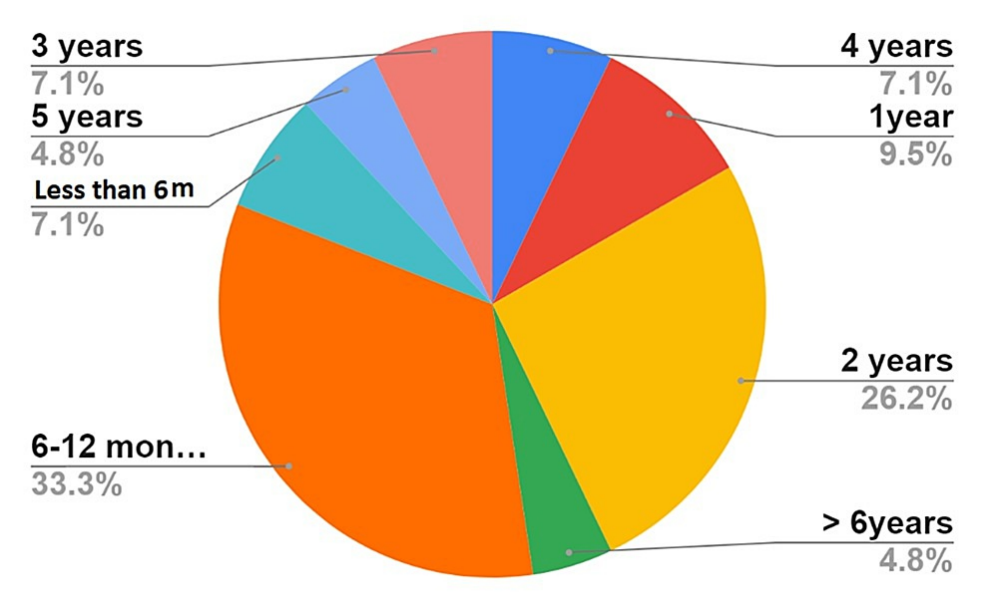
Duration of uveitis diagnosis before starting ADA. ADA, adalimumab

One-third of the patients started ADA between 6 and 12 months after they were diagnosed with uveitis. This percentage dropped to 9.5% one year after diagnosis, only to increase again to 26.2% during the third year. Almost seven percent (7.1%) of the patients started ADA within six months of uveitis diagnosis. Only two patients needed ADA to be started after a period of more than six years after the diagnosis of uveitis (Figure 4).

**Figure 5:**
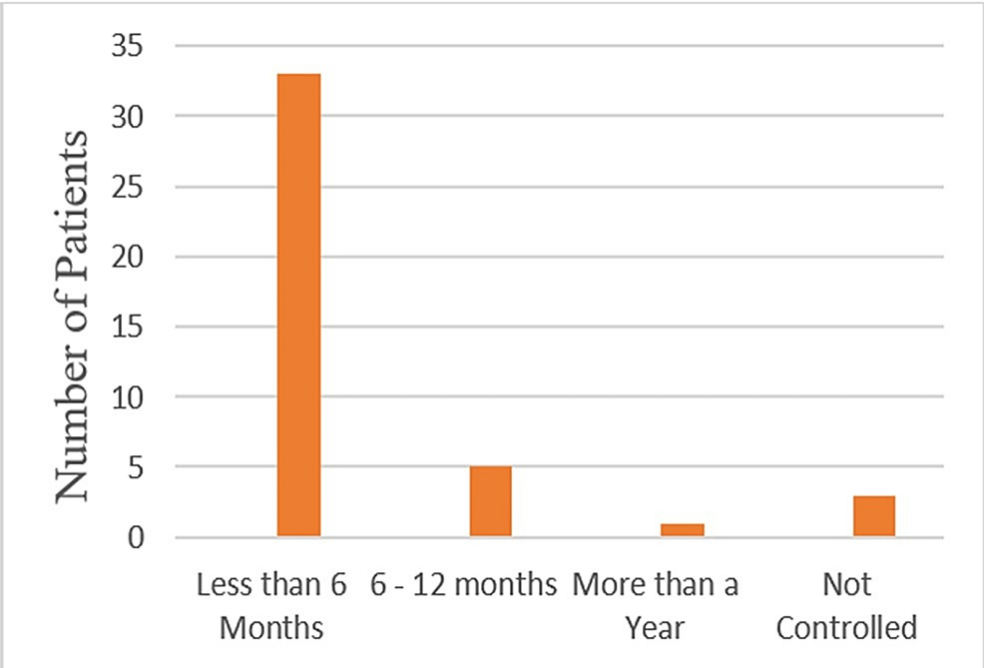
Duration of uveitis diagnosis before starting ADA. ADA, adalimumab

Seventy-eight percent of patients achieved clinical remission with ADA use (no uveitis activity detected, with topical steroid less than two drops a day), while 14.3% were transiently controlled (over more than three months' control before disease relapse). Only 7.1% did not have clinical benefits from ADA (could not achieve disease control, per study definition, within six months). Almost 78.6% showed full response and control of the uveitis inflammation in less than 6 months, while 11.9% needed 6-12 months to achieve disease control (Figure 5). 

Eight patients from the group who were not disease controlled on ADA were tested for anti-drug antibodies, with positive results (more than 10 Au/mL) in three patients (two had a level >200 Au/mL, and one had a level of 168 Au/mL). In one patient, anti-drug antibodies developed after 12 years of ADA use, and in the other, after 4 years. Both were using MTX along with ADA. In the third patient, using ADA monotherapy, positive anti-drug antibodies were detected in the first year of treatment.

The patients did not describe discomfort with the self-administered (carer-administered) injection. Only one patient had a recurrent urinary tract infection. Generally, the group included was satisfied with ADA use.

## Discussion

The proinflammatory cytokine tumor necrosis factor α (TNF-α) is considered a major factor in ocular/uveitis inflammation [3]. Its level in aqueous humor and serum is upregulated in patients with non-infectious uveitis. ADA is a monoclonal antibody against TNF. Several studies have shown that ADA is effective and safe in adult and pediatric patients with NIU, including the landmark VISUAL and SYCAMORE Phase 3 randomized control studies [4-5].

The VISUAL studies described multicenter randomized controlled trials evaluating the efficacy of ADA for adult non-infectious uveitis including patients with various etiologies, mainly Bechet, Sarcoidosis, and Vogt-Koyanagi-Harada disease [1]. Other studies of adolescent and pediatric patients described how safe and effective ADA can be in the management of Bechet disease, ulcerative colitis, and psoriasis [6-9].

The SYCAMORE study, conducted in, described the effectiveness of treatment of juvenile idiopathic arthritis (JIA)-associated uveitis with ADA plus methotrexate (MTX) [5]. Treatment failure occurred in 27% of patients given ADA compared to 60% given a placebo. In our group of patients, we noticed a lower percentage of failure of treatment: 7.1% failure of response, or 14.3% transient control.

Many studies describe the efficacy and safety of ADA with MTX [5, 10-12] for the treatment of NIU in our group; 90% of patients were using MTX as a steroid-sparing agent at baseline and this was continued in the majority of patients, with ADA used as a combination to achieve disease control. Furthermore, MTX, even in low doses, continued to be used to decrease the risk of developing antibodies against ADA. Immunogenicity against biologic drugs is a recognized risk and known cause of treatment failure. Anti-drug antibodies may reduce ADA serum levels and the efficacy of treatment. That can be due to increasing ADA clearance or neutralizing the drug effect, or due to both mechanisms [12-13]. In one study, anti-drug antibodies were found in 16%-26% of ADA-treated JIA patients [12]. Auto-drug antibodies were found less frequently among patients receiving concomitant MTX [12]. In our study, however, we did not test for antidrug antibodies in all patients, and the clinical meaning of positive ADA in patients’ response to treatment is uncertain. This can help to predict the early development of antidrug antibodies in patients using ADA without a cover of another immune modulatory therapy (IMT), mainly MTX, but a larger number needs to be observed to get to the conclusion of the necessity of using an adjuvant IMT with ADA.

Most of the patients in our study group were using topical steroids, and a third of the patients were using systemic steroids before using ADA. However, systemic steroids were reduced to 14% of the cases, with further slow reduction in the majority of the cases started on ADA. A study published in 2018 found that 93% were free of systemic steroids [14] while another study gave a percentage of 54% free of systemic steroids [15]. A study published 10 years ago described that ADA could help in improving vision but could not eliminate the need for systemic or periocular steroids [16], while other studies describe it as a drug that causes steroid-free remission [17].

 In our study, the majority of patients were controlled on ADA in less than a year, while 78.6% were well controlled in less than 6 months. A study published in 2018 found those who started on ADA achieved quiescence at week 78 [10]. On the other hand, 14.3% of our patients relapsed after being well-controlled, while 7.1% did not respond to ADA. A recently published study gave a 49% of relapse. However, the latter study included 26 non-JIA uveitis pediatric patients [18]. The SYCAMORE Study Group published in 2017 found 27% failure of JIA-associated uveitis control with MTX and ADA, compared to 60% of MTX in the placebo group [5].

## Conclusions

This data further support evidence that ADA is an effective and well-tolerated drug in children with refractory uveitis. It can achieve control of ocular inflammation within 6 months of initiation in the majority of patients and achieve steroid-sparing effect. It might also be concluded that the use of DMARDs, especially MTX, is mandatory along with the use of ADA even if good control can be achieved with ADA as a monotherapy. However, a larger number needs to be studied to confirm the second conclusion.

## References

[1] Hasegawa E, Takeda A, Yawata N, Sonoda KH (2019). The effectiveness of adalimumab treatment for non-infectious uveitis. Immunol Med.

[2] LaMattina KC, Goldstein DA (2017). Adalimumab for the treatment of uveitis. Expert Rev Clin Immunol.

[3] Jaffe GJ, Dick AD, Brézin AP (2016). Adalimumab in patients with active noninfectious uveitis. N Engl J Med.

[4] Nguyen QD, Merrill PT, Jaffe GJ (2016). Adalimumab for prevention of uveitic flare in patients with inactive non-infectious uveitis controlled by corticosteroids (VISUAL II): a multicentre, double-masked, randomised, placebo-controlled phase 3 trial. Lancet.

[5] Ramanan AV, Dick AD, Jones AP (2017). Adalimumab plus methotrexate for uveitis in juvenile idiopathic arthritis. N Engl J Med.

[6] Ramanan AV, Dick AD, Jones AP (2019). Adalimumab in combination with methotrexate for refractory uveitis associated with juvenile idiopathic arthritis: a RCT. Health Technol Assess.

[7] Croft NM, Faubion WA Jr, Kugathasan S (2021). Efficacy and safety of adalimumab in paediatric patients with moderate-to-severe ulcerative colitis (ENVISION I): a randomised, controlled, phase 3 study. Lancet Gastroenterol Hepatol.

[8] Kivelevitch D, Menter A (2017). Adalimumab in paediatric psoriasis. Lancet.

[9] Ho M, Chen LJ, Sin HP (2019). Experience of using adalimumab in treating sight-threatening paediatric or adolescent Behcet's disease-related uveitis. J Ophthalmic Inflamm Infect.

[10] Suhler EB, Adán A, Brézin AP (2018). Safety and efficacy of adalimumab in patients with noninfectious uveitis in an ongoing open-label study: VISUAL III. Ophthalmology.

[11] Suhler EB, Jaffe GJ, Fortin E (2021). Long-term safety and efficacy of adalimumab in patients with noninfectious intermediate uveitis, posterior uveitis, or panuveitis. Ophthalmology.

[12] Leinonen ST, Aalto K, Kotaniemi KM (2017). Anti-adalimumab antibodies in juvenile idiopathic arthritis-related uveitis. Clin Exp Rheumatol.

[13] Garcês S, Demengeot J, Benito-Garcia E (2013). The immunogenicity of anti-TNF therapy in immune-mediated inflammatory diseases: a systematic review of the literature with a meta-analysis. Ann Rheum Dis.

[14] Sen ES, Sharma S, Hinchcliffe A, Dick AD, Ramanan AV (2012). Use of adalimumab in refractory non-infectious childhood chronic uveitis: efficacy in ocular disease--a case cohort interventional study. Rheumatology (Oxford).

[15] Hiyama T, Harada Y, Doi T, Kiuchi Y (2019). Early administration of adalimumab for paediatric uveitis due to Behçet's disease. Pediatr Rheumatol Online J.

[16] (2022). Clinical Commissioning Policy: Adalimumab (Humira) and Infliximab (Remicade) as Anti-TNF Alpha Treatment Options for Paediatric Patients with Severe Refractory Uveitis Reference: NHS England D12/P/a. https://www.engage.england.nhs.uk/consultation/specialised-services-consultation/user_uploads/uveitis-paediatrics-policy.pdf.

[17] Castiblanco C, Meese H, Foster CS (2016). Treatment of pediatric uveitis with adalimumab: the MERSI experience. J AAPOS.

[18] Kouwenberg CV, Koopman-Kalinina Ayuso V, de Boer JH (2022). Clinical benefits and potential risks of adalimumab in non-JIA chronic paediatric uveitis. Acta Ophthalmol.

